# Physical Therapy Adjuvants to Promote Optimization of Walking Recovery after Stroke

**DOI:** 10.4061/2011/601416

**Published:** 2011-10-12

**Authors:** Mark G. Bowden, Aaron E. Embry, Chris M. Gregory

**Affiliations:** Rehabilitation Research and Development Service, Ralph H. Johnson VA Medical Center, Department of Health Science and Research and Division of Physical Therapy, Medical University of South Carolina, 77 President Street, MSC 700, Charleston, SC 29425, USA

## Abstract

Stroke commonly results in substantial and persistent deficits in locomotor function. The majority of scientific inquiries have focused on singular intervention approaches, with recent attention given to task specific therapies. We propose that measurement should indicate the most critical limiting factor(s) to be addressed and that a combination of adjuvant treatments individualized to target accompanying impairment(s) will result in the greatest improvements in locomotor function. We explore training to improve walking performance by addressing a combination of: (1) walking specific motor control; (2) dynamic balance; (3) cardiorespiratory fitness and (4) muscle strength and put forward a theoretical framework to maximize the functional benefits of these strategies as physical adjuvants. The extent to which any of these impairments contribute to locomotor dysfunction is dependent on the individual and will undoubtedly change throughout the rehabilitation intervention. Thus, the ability to identify and measure the relative contributions of these elements will allow for identification of a primary intervention as well as prescription of additional adjuvant approaches. Importantly, we highlight the need for future studies as appropriate dosing of each of these elements is contingent on improving the capacity to measure each element and to titrate the contribution of each to optimal walking performance.

## 1. Introduction

Cerebrovascular accident (CVA) is one of the leading causes of long-term disability in the United States [1]. There are approximately 795,000 CVAs per year and currently 6.5 million noninstitutionalized stroke survivors in the United States [1], with increased survivorship due to improved interventions [2, 3]. Recent estimates report approximately one-half of survivors regain ambulation [4], with at least a minimal degree of gait impairment [5]. Returning to prior level of function, most importantly independent ambulation, remains a priority to stroke survivors [6, 7]. This demand from patients as well as the fact that locomotor ability is an important factor in determining level of disability [4] has led to an increased focus on physical therapy interventions to improve walking performance. 

Advancement of interventions, particularly those which target available neuroplasticity and purport to aid in neuromotor recovery (as opposed to teaching compensatory strategies), is limited by a paucity of informative outcome measures. Self-selected walking speed is an important measure of stroke rehabilitation because it is simple to measure, reflects both functional and physiological changes [4, 8], remains reliable and sensitive to change even as recovery advances [9], and is a predictor of health status [10] as well as quality of life [8]. Walking speed has been used often in locomotor rehabilitation for individuals post-stroke for interventions such as exercise therapy [11], lower extremity strength training [12, 13], functional electrical stimulation [14], treadmill walking [15, 16]; and locomotor training with treadmill and body weight support [17–19]. However, changes in walking speed are not statistically different between these intervention approaches, leading some to advocate that clinical decision-making should be guided by a “pragmatic approach” [20]. The lack of superiority of one treatment approach over another may be related to the limitations of contemporary outcome measurement to provide information about mechanisms of response to treatment or to assist with dosing of constituent treatment elements. In addition, stroke is a very heterogeneous phenomenon, and treating a sample of convenience with a single therapeutic approach dilutes the potential benefits to those for whom the approach may be most appropriate. Given the multifaceted impairments and activity limitations in those after stroke, it is likely that a singular approach only targets an individual limiting component, and that investigations into combinations of interventions are required to maximize ambulatory potential.

In this perspective paper, we propose a different view of the concept of adjunctive therapies and posit that walking performance is limited by a combination of impairments in the following physical characteristics: (1) walking specific motor control, (2) cardiorespiratory fitness, (3) dynamic balance, and (4) muscular strength. Traditionally, an adjuvant therapy is defined as an agent added to a universally accepted treatment approach to increase its effectiveness. In locomotor rehabilitation, however, there is currently no universally accepted treatment, and many different treatments to improve walking after stroke result in similar effects. Thus we contend that there is no single therapy to which adjuvants should be added, but rather evaluation using proper measurement tools should dictate which treatment is most critical and which others should be added in the proper doses. In addition, a critical component to these interventions is that they must be of the appropriate intensity, frequency, and duration to maximize the capacity for neuromuscular plasticity and functional change. Importantly, these four factors are all amenable to physical therapy intervention and have been associated with similar improvements in locomotor outcomes. Singular approaches should be accompanied by some combination of these adjunctive rehabilitation interventions, and future medical treatments (e.g., pharmacological or surgical approaches) should be investigated not only in concert with singular interventions, but with the composite program designed to address the existing physical impairments and facilitate recovery of locomotion. In this paper, we provide a background for each of these four approaches, describe the outcomes of treatment studies incorporating these approaches, present measurement techniques most often utilized, and address the need for exploration of underlying mechanisms to task accomplishment that are amenable to change using any single or combination of the targeted physical therapy interventions. We fully recognize that there are likely additional limiting factors (e.g., altered sensation, cognitive function, and personal/environmental variables) that also contribute to functional recovery; however, the four factors mentioned above are most commonly addressed with physical therapy intervention.

## 2. Walking-Specific Motor Control

### 2.1. Theoretical Framework

Human walking is an incredibly complex task involving a multitude of degrees of freedom and a large number of combinations of muscle activation patterns. While it is perhaps staggering to consider that the brain can voluntarily control all of these variables during a cyclic, rhythmical task such as walking, definitive studies of the spinal control of walking in humans are difficult to conduct due to the inability to directly assay the human central nervous system. In addition, modifications that humans must make to meet the demands of upright bipedalism make direct translation from studies of quadruped pattern generation exceedingly problematic [21]. However, we may surmise from human studies the complex interaction that encompasses walking-specific motor control. As early as 1997, Harkema et al. demonstrated that in individuals with clinically complete spinal cord injury, the neuronal mechanisms within the lumbosacral spinal cord are able to “modulate efferent output in a manner that may facilitate the generation of stepping” [22]. More recent work with those with incomplete spinal cord injury demonstrate improvement in the integrity of descending corticospinal pathways as manifested with improved motor evoked potential after therapy [23]. As Nielsen stated in a review of the central control of muscle activity during walking, “it is the task of the whole central nervous system to generate (this) muscle activity, to ensure that it is optimally coordinated, to ensure that it is adjusted to the immediate environment, and to modify it when required” [21]. Nielsen concludes by saying that there is “no reason” to suggest that human walking is controlled exclusively by the spinal cord, nor is there a reason to imply that the motor cortex alone is responsible for activation of muscles during walking. Instead, this activity related to walking must rely on an integration of spinal neuronal circuitry, afferent signals, and descending motor commands [21]. As a result, treatments designed to access and improve available motor control need to not only target descending volitional neural drive, but also provide afferent input that increases the potential for subcortical neural networks to modulate appropriate efferent output.

### 2.2. Treatment

Interventions that focus on increasing activity in impaired body segments, as opposed to focusing on compensatory strategies, are critical for training all aspects of the neuromotor axis. Repetitive step training, on a treadmill with or without body weight support, or over-ground, is a modality for locomotor rehabilitation that allows intense practice with the maximal loading that the person can support on their lower extremities while facilitating upright trunk posture, hip extension, and proper lower limb loading and unloading at normal walking speeds. Repetitive step training provides sensory inputs specific to walking and allows effective lower limb stepping practice [24, 25] based on the principles of activity-dependent plasticity. This approach allows for mass practice of coordinated stepping, and thus may provide an opportunity to alter motor control during walking. While effective by itself in improving walking-related outcome, two recent clinical practice guidelines [26, 27] state that, at present, the data are inconclusive to distinguish repetitive step training from other therapies, and the recent results from the LEAPS study show equal improvement with progressive home exercise strengthening programs [19]. To date, no study has utilized repetitive step training as the primary intervention with appropriately intense adjuvant therapies, nor utilized this training as an adjuvant to another physical intervention. It is likely that focusing purely on the ability to increase stepping capacity may ignore other critical factors limiting the overall efficacy of the locomotor rehabilitation program.

### 2.3. Measurement

Historically, measurement of motor control after stroke has been defined by the concept of progressing through predictable stages of recovery [28, 29]. Based on this theory, Fugl Meyer et al. [30] developed a measurement instrument reflecting this reflex hierarchy to quantify motor recovery after stroke [30]. The FM-LE, however, consists of voluntary, discrete tasks based on the dominant influence of cortical input on motor control and examines motor control in three theoretically progressive positions: supine, sitting, and standing. However, the motor control deficits that the FM measures may differ from deficits seen during task-specific activities such as walking. Neckel et al. investigated the abnormal movement patterns seen after stroke by analyzing strength deficits and movement patterns from a “functionally relevant” standing position [31] and found that those with stroke were significantly weaker than neurologically healthy control subjects. Those with hemiplegia and controls used similar strategies to achieve movements, and only during maximal hip abduction did a significant secondary movement of hip flexion emerge in those with hemiplegia, mimicking the abnormal synergy patterns described by Brunnstrom et al. [29] and Fugl Meyer [30]. These results suggest that the primary impairment in poststroke motor control is weakness, and that correct interpretation of poststroke motor control can only be gleaned from positioning that is relevant to the targeted behavior. Voluntary, discrete activities may be inadequate to capture the complex motor behavior in walking. In contrast, walking-specific measures may best describe the effect of walking rehabilitation interventions. These walking-specific measures need to go beyond commonly used walking speed, and address the underlying biomechanical mechanisms and quality of movement during functional outcomes. We have demonstrated in previous work that paretic propulsion (defined as the percentage of the total positive impulse (propulsion) generated by the paretic leg) is an indicator of hemiparetic severity [32] and is such a task-specific measurement. Additionally, the paretic step ratio (defined as the percentage of the stride length accounted for by the paretic step length) is highly correlated with paretic propulsion and can serve as a clinical analogue when kinetic data are not available [33]. While no definitive measure exists at this time, it is critical to begin to move beyond voluntary and isolated clinical exams to describe walking-specific motor control.

## 3. Cardiorespiratory Fitness

### 3.1. Theoretical Framework

Persons after stroke typically present with profound deconditioning, reflected in measures of cardiorespiratory fitness ~50% below age-matched control subjects [34, 35]. This level of deconditioning, in combination with an increased energetic demand of walking, likely limits locomotor performance during activities of daily living. Specifically, metabolic economy, expressed as oxygen consumption normalized to walking speed, is approximately 50% higher in persons after stroke relative to controls [34]. This, combined with the aforementioned reduction in maximal oxygen consumption, results in an extremely high relative intensity of activity, likely limiting walking to very short distances in most cases. 

The physiological mechanisms that contribute to decreased fitness post-stroke include alterations in motor control, reduced peripheral muscle activation, atrophy of peripheral muscle, a shift in muscle molecular phenotype, reduced oxidative enzyme activities, and altered peripheral hemodynamics. Given that stroke is a condition commonly associated with advanced age, these changes combine to increase disability that is further accelerated by sarcopenia and fitness decrements associated with advanced age [36, 37]. These characteristics are responsive to exercise and thus represent critical targets for rehabilitation intervention. Hence, substantial emerging evidence suggests that exercise can improve cardiorespiratory fitness, even years after stroke. Epidemiological evidence further suggests that the reduced cardiovascular fitness and the secondary biological changes in peripheral muscle following stroke may propagate components of metabolic syndrome, conferring an added morbidity and mortality risk [35]. Taken together, these benefits emphasize the need for development of training paradigms that offer a multisystems approach to improving both neurological and cardiovascular health outcomes in the poststroke population.

### 3.2. Treatment

Aerobic conditioning can be valuable to persons following stroke by improving functional ability, reducing the risk of recurrent morbid events, and improving overall quality of life. Improvements in cardiorespiratory fitness are commonly achieved by the systematic approach of aerobic training. This training can be performed in a variety of methods (i.e., recumbent bike, arm bike, treadmill walking, stair climbing, etc.) with the common goal of improving the body's ability to consume oxygen during physical activity. Recent studies demonstrate the effectiveness of aerobic exercise training for improving cardiorespiratory fitness in persons after stroke [38, 39]. There are also indications that activity-level functions such as locomotor ability may also be enhanced through such programs. For example, in a recent meta-analysis, Pang demonstrated an overall positive effect of treadmill aerobic exercise training that was independent of the stage of stroke recovery, the key characteristic of training being the intensity of treadmill exercise [40]. The findings of this analysis also show that improvement in VO_2peak_ following treadmill aerobic exercise is accompanied by improved walking velocity and walking endurance, emphasizing aerobic exercise as a critical component of stroke rehabilitation. However, to date studies provide little data concerning the effects of dose of aerobic exercise (i.e., frequency, intensity, duration, and mode) on physiological or functional outcomes in this population. Moreover, the potential influence of combining aerobic-type exercise with strengthening, balance, or motor control therapies is yet unknown. Because of the common comorbidities present in the poststroke population (i.e., hypertension, dyslipidemia, obesity, and depression), specific recommendations for prescribing aerobic exercise must consider other modalities in the treatment regimen of this large and diverse population. Future research must address appropriate design of cardiorespiratory fitness programs necessary to maximize gains in functional ability as well as reductions in cardiovascular risk factors in persons following stroke.

### 3.3. Measurement

Cardiorespiratory fitness relates to an individual's ability to perform prolonged physical activity and is commonly expressed as the maximal volume of oxygen consumed (VO_2max_) during steady-state exercise. This value represents the capacity of the circulatory and respiratory systems to supply oxygen and of peripheral skeletal muscle to utilize oxygen during task performance. Although measures of cardiorespiratory fitness are valid, reliable, and relatively easy to collect in a research setting, some factors must be considered when performing such measures in the poststroke population. For example, the mode of exercise used in the poststroke population will be dependent on the ability of the individual to perform high-intensity exercise. Treadmill exercise will not be appropriate for individuals with limited ability to stand or walk at fast speeds or up an incline, while upright cycling can be complicated by an inability to utilize handle bars in those with severely impaired upper extremity function. Cycling is also difficult in subjects with profound difficulties performing the bilateral reciprocal muscle actions required for pedaling a cycle ergometer. As such, exercise capacity may be limited (somewhat) by the mode of exercise chosen for testing in this population. Thus, the most appropriate outcome measure to report will be the peak oxygen consumption (VO_2peak_) measured during exercise, as the historical indicator of true maximal oxygen consumption (e.g., plateau in VO_2_ with increasing exercise intensity) will likely not be elicited in this population. In addition, considerations for medical histories that may limit the physiological responses (e.g., increases in blood pressure and heart rate) achieved in response to both maximal and submaximal exercise must also be made. As in all exercise studies, it is critical to utilize the same mode of exercise as well as the same protocol for measuring cardiorespiratory fitness when performing repeated assessments. 

## 4. Dynamic Balance Control

### 4.1. Theoretical Framework

**Figure 1:**
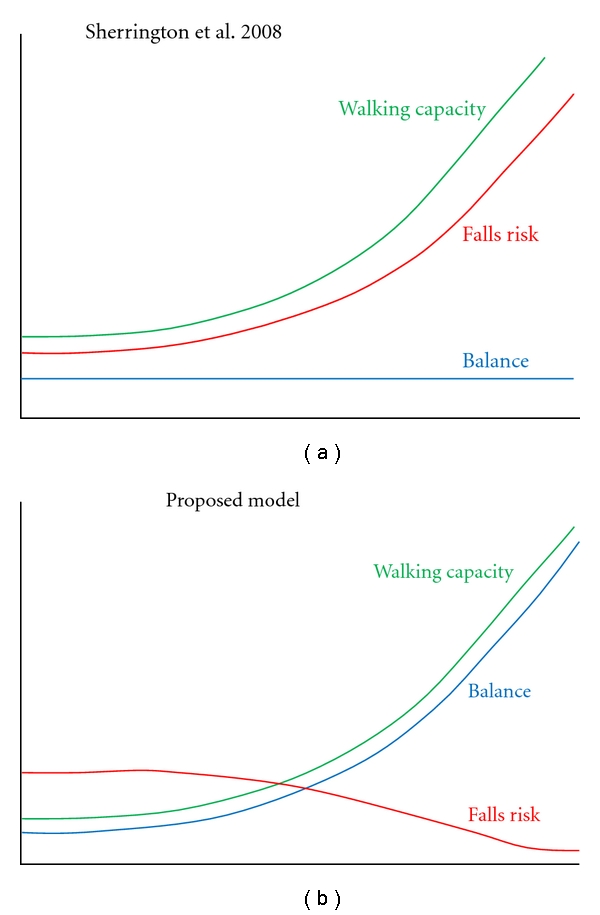
(a) Illustration of Sherrington et al.'s [41] conclusion of increased risk of falls if no balance intervention is supplied and walking capacity increases and (b) a theoretical depiction of combined effects of walking capacity with balance training on falls risk.

Maintaining balance in the most basic sense is defined as not falling. The consequences of falling are well documented and risk factors have previously been identified [42]. The rationale for rehabilitation to improve balance and thus reduce these risks is clear. Recent reports of walking rehabilitation programs have investigated the effects on clinical measures of balance control [43] and have found that locomotor rehabilitation can positively influence clinical measures of balance. However, Sherrington et al. reported that exercise programs that do not contain an aspect of walking may result in a decreased risk of falls among the ambulatory population with balance difficulties (Figure 1(a)) [41]. A potential explanation for this finding is tied to activity risk exposure and its relationship to functional impairment status. Balance recovery is critical to improving functional walking status and solely retraining stepping capacity may in fact limit functional independence by subsequently increasing falls risk. Conversely, if dynamic balance control is trained concurrently with stepping capacity, functional walking performance may increase simultaneously, thereby decreasing risk of falling (Figure 1(b)). 

Neurological injury, such as a stroke, not only limits walking performance, but also may result in a reduction in balance due to a disruption in the precise communication of afferent and efferent information to control and refine movement. Subsequent reduction in physical performance results in reduced fitness levels and impaired strength of involved body segments making it very difficult to distinguish impaired dynamic balance from deconditioning, reduced motor control, and decreased muscle strength. The importance of clearly defining balance for measurement and training is imperative but is limited by a lack of consensus on how to incorporate dynamic tasks in the quantitative and qualitative balance reporting of neurologically impaired individuals.

### 4.2. Treatment

Balance training has been shown to be beneficial in the elderly population [44] as well as in stroke survivors [45]. Improving balance as it relates to gait and mobility is a common and important goals for physical therapists when working with participants following a stroke. With the large heterogeneity of physical capabilities among stroke survivors across different settings, no one single intervention has been proven most beneficial for optimizing balance outcomes [44, 45]. Some general exercise principles exist in the balance training literature, and relate to increasing task complexity and difficulty as participants demonstrate improved physical function and balance capabilities [45]. There is little to no investigation of the use of higher level balance tasks utilized in the athletic training literature in the stroke cohort, although this may be an appropriate and perhaps required intervention to maximize walking recovery. Frequency and dosing of the intervention is widely variable with the only limiting factor in many studies being attrition due to tolerance [44–46]. Costello et al. report that a minimum of 12 weeks intervention duration is necessary to see meaningful balance improvements [44], but subsequent reviews report paucity in this number [45]. One significant variable of basic exercise principles that is often overlooked is the intensity of the intervention. With significant concern placed on patient fall risk and individual task success, balance is difficult to challenge without the utilization of expensive safety equipment. A delicate interplay between type of intervention and applying the optimal intensity is difficult with balance training in the neurologically impaired population. Measurement of task intensity is also difficult and has not been adequately quantified. Significant concern to protect those involved in the training against injury often prohibits “successful failure” that may promote unimpeded improvement of balance reactions [47]. Providing the proper intensity for balance and functional carryover is important to achieve optimal outcomes [48]. A conceptual framework of individualized functional tasks directly related to gait, to train both central and peripheral neuromuscular processes, is perhaps the most appropriate intervention. Translating this concept into realistic and clinically feasible interventions is an evolving science and relies heavily on clinically appropriate and accurate measurement tools to assess change.

### 4.3. Measurement

As an outcome measure, balance is commonly based on a quantitatively recorded number of falls, which is then qualified as having some degree of balance (i.e., good/fair/poor) or being at a predefined relative risk for falls. Alternate definitions are more biomechanical in nature and define balance as the ability to maintain the center of mass within the base of support or the control of angular momentum [49]. These definitions require kinetic and kinematic measurement utilizing expensive equipment usually reserved for laboratory settings and have limited functional significance to the clinician. Balance has been subdivided into four identifiable conditions: (1) static postural control, (2) postural control with voluntary actions, (3) postural control with involuntary actions, and (4) postural control during external perturbations [50]. These four subdivisions theoretically encompass static and dynamic activities including walking. It is important to draw a clear distinction to the task of walking as it relates to balance from static standing, due to the significant differences in mechanisms governing static and dynamic conditions [51]. However, it is difficult to assess balance impairments during walking in persons with neurological deficits because the assessment of dynamic balance during walking in healthy persons is still developing. As such, dynamic balance control remains largely unexplored, though it is increasingly recognized as a critical feature in understanding the comprehensive construct of functional walking performance. In addition, more information is needed regarding dynamic balance control as it relates to the multiple dimensions of functional walking in addition to steady-state activities [52]. 

As balance relates to walking performance there are few measurement tools successfully utilized in practice and research that adequately demonstrate underlying neuromotor capabilities and changes following intervention or injury. The Berg Balance Test (BBT) is the most commonly utilized balance tool [53], and although the BBT demonstrates excellent psychometric properties, including predictive validity [54], it has not been shown to be the best determinant of walking recovery and function [55]. Its predominant use in research perhaps represents the lack of viable alternatives and an opportunity to improve the measurement tools for measuring balance as it relates to gait and walking function. Other less frequently utilized functional performance measures to quantify balance capabilities are the Timed Up and Go test (TUG) or the Dynamic Gait Index (and its modified form, the Functional Gait Assessment). All have been shown to be reliable in the stroke population [56, 57], but fail to describe the mechanistic underpinnings of impaired balance which are required to formulate the most effective treatment plan. Force plate analysis has been successfully utilized in laboratory settings to demonstrate postural control and balance changes noted after a stroke [58, 59], but are not utilized in clinical practice settings and do not relate directly to dynamic balance control during walking. A clinically applicable and feasible balance tool that properly assesses the neuromotor mechanisms is needed to guide clinical decision making regarding the most effective treatment approaches as well as monitor neuromotor recovery.

## 5. Muscular Strength

### 5.1. Theoretical Framework

The most common motor consequence following stroke is hemiparesis, defined as (muscular) weakness on one side of the body. It is estimated that over 65% of stroke survivors experience hemiparetic motor dysfunction up to 1 year after stroke [60]. Collectively, contemporary research findings demonstrate that weakness is a major contributor to functional disability following stroke, with impairments in muscle strength associated with decreased independence in activities of daily living (ADL). The theoretical framework underlying the importance of muscle weakness and function is straightforward: force equals mass times acceleration (*F* = ma). Consequently, acceleration of the mass of the body (or individual body segment) requires force generation by skeletal muscles. To the extent that stroke impairs skeletal muscle force generation, acceleration will be limited and functional performance compromised. Beyond the theoretical, functional capacity is extremely limited in many persons after stroke and muscle weakness is an impairment that is responsive to rehabilitation intervention.

**Figure 2:**
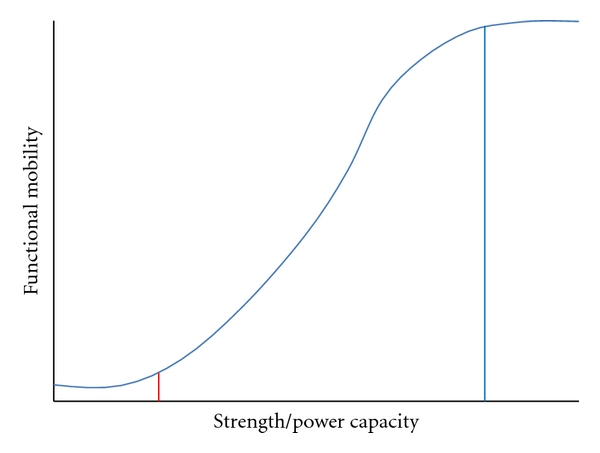
Theoretical association between muscle strength and functional mobility.

Muscular weakness following stroke involves both direct (i.e., damage to neural structures) and indirect (i.e., muscular disuse subsequent to reduced physical activity) mechanisms that impact both the ability of the central nervous system to voluntarily activate skeletal muscles as well the force generating capacity of muscle (e.g., atrophy). It is important to recognize that the degree to which muscle weakness impacts functional ability varies greatly and is dependent on task demands. For any given activity, at least conceptually, increases in strength will not be beneficial until a certain threshold is reached (Figure 2, red line). Once achieved, increases in strength will be accompanied by improved functional performance, at least to a certain point (Figure 2, blue line). Thereafter, further increases in strength would not result in functional gains. Importantly, given that functional activities differ in the demands placed on skeletal muscle(s) the point at which force production begins (and ceases) to affect functional performance will vary accordingly, emphasizing the potential importance of muscular strength as a foundation for rehabilitation intervention while, at the same time, highlighting the limits to which strengthening alone can maximize functional performance. 

### 5.2. Treatment

Progressive resistance training (PRT) is widely accepted as the most effective method for developing muscular strength and is currently prescribed by most major health organizations for improving health and fitness. PRT can improve lower extremity strength following stroke and, when delivered at appropriate intensities, can provide significant functional benefit. A recent quantitative review [60] concluded that even though prevailing clinical thought argues that functional improvements emerge only from task-specific training, measurable gains in lower extremity strength following resistance training were associated with functional improvements in a poststroke population. It should be noted that studies describing functional outcomes following strengthening in the poststroke population are historically equivocal, though consistency with regards to intensity of the intervention in recent studies has supported the argument for strengthening this cohort.

Given the clinical presentation of hemiparesis, rehabilitation protocols have often focused on attenuating deficits in paretic musculature. However, it should be recognized that strength of the non-paretic limb is also impaired following stroke and may directly impact functional performance in some tasks. For demanding functional activities requiring the engagement of muscles on both sides of the body (e.g., walking), strengthening of nonparetic muscles may be important for inducing significant functional improvement. In addition, muscles of the trunk can also be impaired after stroke and should not be overlooked when a strengthening regimen is initiated. Interestingly, programs that combine PRT with balance training, and/or aerobic conditioning demonstrate significantly greater improvements in walking function than the individual programs alone [61, 62], providing experimental evidence as to the benefits and limitations of strengthening alone.

### 5.3. Measurement

Clinical assessments of muscle strength in persons after stroke are typically performed using manual muscle tests. Manual muscle testing is based on a 6-point ordinal scale that grades strength from none to seemingly normal. However, manual muscle tests are subject to a ceiling effect, lack sensitivity to change, and have a relatively poor inter-rater reliability [63, 64]. Importantly, manual muscle tests lack the ability to evaluate the temporal characteristics of muscle performance, which have been reported to be important predictors of function in various populations, including stroke [65–67]. Given the inherent limitations of manual muscle testing, more sensitive means of quantifying force production are necessary to accurately measure muscle strength after stroke. Commercially available dynamometers have increased sensitivity as well as the ability to capture temporal characteristics associated with voluntary force production, making these devices attractive alternatives to manual muscle testing. The most common outcomes of dynamometric strength testing are maximum voluntary isometric contraction (MVIC) as well as peak concentric and eccentric torque generation at various criterion speeds measured during isokinetic testing. The ability to asses force production during different muscle actions (i.e., concentric versus isometric versus eccentric) and across a range of contractile velocities makes measurement of muscle strength a valuable outcome measure for use during rehabilitation research studies in the poststroke population. In addition, dynamometric data can be simultaneously acquired with other measures (e.g., EMG or superimposed electrical stimulation) that provide added information about the mechanisms underlying deficits in muscle strength as well as the response to intervention.

## 6. Conclusion

**Figure 3:**
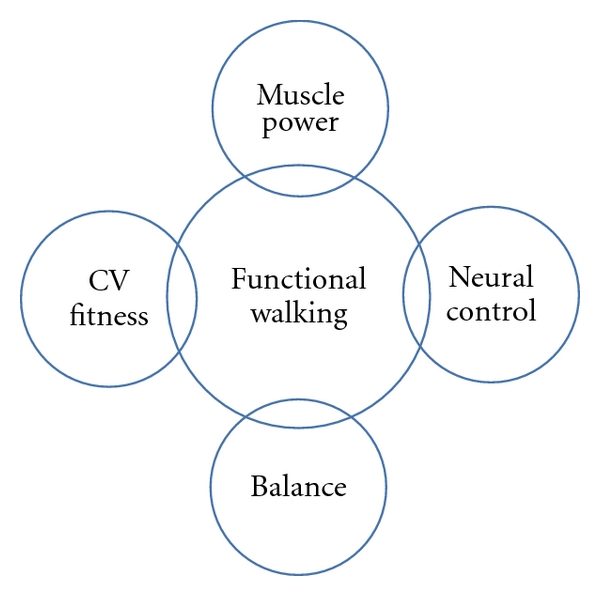
Walking performance is likely composed of four main physical components (cardiorespiratory fitness, strength, motor control, and dynamic balance) with additional unknown contributing factors. The degree of contribution and overlap is unknown at this time and part of the individualized nature of poststroke locomotor dysfunction.

Functional walking performance cannot be fully addressed by a singular intervention. Research, by necessity, tends to investigate singular interventions, but progression towards the optimal strategy to retrain walking after stroke demands that investigations begin to include elements targeting multiple areas of deficit. We propose a multidimensional framework within which walking recovery following a stroke can focus on the combined utilization of a primary intervention approach (e.g., one identified as the most significant contributor to functional limitation) with one or more of the interventions presented herein (Figure 3).

The intervention needs to be tailored to the patient's deficits and chosen based on the most appropriate measurement tools available. In the proposed model, measurement will prove to be critical in dosing specific elements as part of the individualized determination of need. Furthermore, each element must be addressed with therapeutic strategies of appropriate intensity, frequency, which are demonstrated in several recent reviews to be critical elements of successful locomotor rehabilitation [20, 68]. In addition, these parameters are identified as part of a set of core principles that are critical to maximizing neural plasticity after brain damage [69].

We are not the first to advocate a multi-dimensional approach to locomotor rehabilitation, and other investigators have advocated similar strategies for recovery of walking following stroke [70, 71]. Intensity is well understood and utilized in the athletic training literature, but there is questionable carryover into clinical neurorehabilitation. In the case of stroke, the disease process directly affects the neurological, musculoskeletal, and cardiovascular systems, with significant secondary sequelae resulting in impaired motor function. The stroke survivor demographic is generally older with reduced fitness levels, and higher risks for cardiovascular diseases and comorbidities. It is not only imperative that this cohort is subjected to proper intensity and specificity of therapies targeting recovery of walking, but also for the prevention of common comorbidities associated with increased age and decreased activity levels. The model presented herein aims to expand earlier task-oriented strategies through individualized dosing and systematically prescribed increases in intensity.

In this perspective paper, we explore the concept of a multidimensional approach to physical therapy following a stroke. We advocate utilizing a variety of potential physical adjunctive therapies to optimize walking outcomes, any one of which might be the primary therapy as determined by appropriate measurement. This concept remains somewhat theoretical as the field of locomotor rehabilitation after stroke presently lacks adequate measurement tools for stepping capacity and balance which would guide clinical decision making. As such, part of this perspective paper is speculative and identifies areas in which future research is needed to guide this process. However, this framework could serve as a future clinical decision making guideline or infer a more targeted and systematic method for training walking recovery. We present this theoretical framework to direct and challenge research to move beyond the model of single therapeutic intervention model and into a more complex but promising paradigm for walking recovery research and treatment.
